# Quantitative Evaluation of the Use of Actigraphy for Neurological and Psychiatric Disorders

**DOI:** 10.1155/2014/897282

**Published:** 2014-08-19

**Authors:** Weidong Pan, Yu Song, Shin Kwak, Sohei Yoshida, Yoshiharu Yamamoto

**Affiliations:** ^1^Department of Neurology, Shuguang Hospital Affiliated to Shanghai University of Traditional Chinese Medicine, Shanghai 201203, China; ^2^Department of Neurology, Graduate School of Medicine, The University of Tokyo, Tokyo 113-8655, Japan; ^3^Center for Disease Biology and Integrative Medicine, Graduate School of Medicine, The University of Tokyo, Tokyo 113-0033, Japan; ^4^Department of Neurology, Kansai University Clinic, Kansai University of Health Sciences, Osaka 590-0482, Japan; ^5^Educational Physiology Laboratory, Graduate School of Education, The University of Tokyo, Tokyo 113-0033, Japan

## Abstract

Quantitative and objective evaluation of disease severity and/or drug effect is necessary in clinical practice. Wearable accelerometers such as an actigraph enable long-term recording of a patient's movement during activities and they can be used for quantitative assessment of symptoms due to various diseases. We reviewed some applications of actigraphy with analytical methods that are sufficiently sensitive and reliable to determine the severity of diseases and disorders such as motor and nonmotor disorders like Parkinson's disease, sleep disorders, depression, behavioral and psychological symptoms of dementia (BPSD) for vascular dementia (VD), seasonal affective disorder (SAD), and stroke, as well as the effects of drugs used to treat them. We believe it is possible to develop analytical methods to assess more neurological or psychopathic disorders using actigraphy records.

## 1. Introduction

Some neurological and psychopathic diseases such as Parkinson's disease (PD), cerebral infarction (CI), seasonal affective disorder (SAD), restless leg syndrome (RLS), vascular dementia (VD), Alzheimer's disease (AD), and even sleep disorders and depression are related to abnormal activity symptoms. It is necessary to evaluate their severity or the effects of drugs to treat these diseases in clinical practice. Although there are many clinical scales for their assessment by physicians or technicians, these scales may not adequately and quantitatively reflect the disease severity. Most of these evaluative measures are subjective or partially objective methods [1]. A reliable objective scale representing disease severity is necessary for appropriate management of various disorders. There are some motion transducer systems available which could be used for long-term monitoring of human motion, including surface EMG long-term recording or wireless systems integrating triaxial accelerometers and gyroscopes [2–5]. Wearable accelerometers (such as an actigraphy AMI, Ambulatory Monitors Inc., USA) enable long-term recording of a patient's movement during activities of daily living [6, 7] and, hence, might be the best choice for a device for quantitative assessment of the symptoms due to various diseases [8]. Recently, studies have been successful in developing reliable analytical methods that quantitatively represent the disease progression in patients with PD, VD, SAD, tremor, and sleep disturbance [9–11]. Here, we review some applications of using actigraphy with analytical methods that are sufficiently sensitive and reliable to determine the severity of these diseases and correlated disorders and the effects of drugs on these disorders. The severity of these disorders might be correlated with physical activity.

## 2. Quantitative Record Device and Analytical Methods

Actigraphy (an accelerometer, also called actigraph or actigram) is a small, custom wrist watch-sized activity monitor equipped with a computer that can register and quantify human physical activity. In its zero-crossing mode (ZCM), the zero-crossing counts are integrated over 1-minute intervals and the data is stored in the internal memory. This activity monitoring device is analogous in performance to the commercial Actigraph Mini-Motionlogger (Ambulatory Monitors, Ardsley, NY) which has frequently been used for studies of physical activity. It has 20 types of recording modes for different sensitivities and specificities with series filter ranges and sensitive thresholds. In most neurological studies, they record activity in the ZCM with a setting comparable to mode 13 of the Mini-Motionlogger (filter range of acceleration signals: 2-3 Hz, sensitive threshold: high, and gain: low) for evaluation of movement disorders [1, 12, 13]. After recording, data are transmitted to an external computer by software installed on the device.

Initially, accelerometry was used for recording general activities and evaluating the ability of movement, such as the mean values of activity. During sleep, it might indicate the approximate activity level or account of the body [14], but, in other situations, activity might be easily influenced by lifestyle or work for diurnal recordings. Matsumoto et al. used accelerometry quantitatively to evaluate essential tremor [15] and Van Someren et al. used it to evaluate long-term tremor recording [6]. Recently, people used body activities translation analysis changefully. In previous studies, after long-term recording, the data were transmitted to a computer and the activity scores were plotted to demonstrate the daily profiles and biological rhythms [16–18]. The data acquired from the actigraphy were categorized into diurnal activity (DA, between 6 a.m. and 6 p.m.), evening activity (EA, between 6 p.m. and 9 p.m.), and nocturnal activity (NA, between 9 p.m. and 6 a.m.) to quantitatively assess activity ability and quality. Furthermore, researchers have also analyzed the activity levels and determined sleep latency (SL), sleep efficiency (SE), awake times, awake time duration, and the least 5-hour activity (5 h) to quantitatively assess sleep quality [16, 17].

Fractal analysis [19, 20] was shown to be a robust tool to disclose hidden autocorrelation patterns in biological data, such as heartbeat and limb movement [21–23]. One type of fractal analysis, detrended fluctuation analysis (DFA), has been used for random-walk analysis, with a recent modification for various “real-world” signals including activity time series [24]. The original DFA evaluates the relationships between time scales and the magnitudes of fluctuation (standard deviations) within each time scale. More correlated signals represent a greater growth of the fluctuation magnitude with increasing time scale or length of data window. It also eliminates the magnitudes of fluctuation and is suitable for the analysis of long-term data collected in daily activity. “Scale-invariant patterns” [25] in activity means that the pattern of fluctuations in activity does not have any periodic oscillations but shows self-similarity over all scales. Fluctuations with self-similarity look similar to each other, whether one extracts slow fluctuations over long periods or rapid fluctuations during short periods. According to this property, researchers use a single parameter called a power law exponent (PLE) developed from DFA to characterize the pattern of fluctuations [1]. This exponent relates to the rapidness of the activity transitions, such as abrupt onsets and offsets, or gradual changes in movements. The PLE, obtained as the slope of a straight line fit in the double-logarithmic plot of time scales versus magnitudes of fluctuation, was used to characterize the level of such correlation. This index reflects the probability of a simultaneous increase or decrease in the variability at two distant points in time in the time series, applied to all distances up to long-range time scales, thereby probing the nature of “switching” patterns between high and low values in a statistical sense. Larger power law exponents indicate positive temporal autocorrelation or persistency in the increase or decrease, and lower values correspond to negative autocorrelation or antipersistency. Its local maxima or minima of fluctuations of locomotor activity would be the most useful for quantitative purposes, as they represent the level of persistency of movement patterns. Pan et al. [1] found that the PLE for local maxima most sensitively and reliably reflects disability of movement disorder for patients with PD without being influenced by the presence of tremor or the patterns of daily living. The advantages of the method related to a wavelet-based power law temporal analysis facilitate the detection of transient variations in detrended records at different time scales.

There are some disadvantages commonly related to the use of DFA for time series variability analysis, such as a clear shift in the state of a biological signal (e.g., spontaneous atrial fibrillation in the ECG signal) or clear sustained artifacts would prohibit meaningful DFA interpretation; the calculation of two scaling exponents may represent a somewhat arbitrary manipulation of the results of the analysis, and the analytic technique is based on this assumption. If the same scaling pattern is present throughout all signals (no fluctuation), the scaling exponents could not be demonstrated.

## 3. Use in Evaluating the Severities of Movement and Sleep Disorders for PD

PD is a neurodegenerative disorder with respect to not only motor symptoms, including resting tremor, rigidity, bradykinesia, and postural instability, but also nonmotor symptoms, including autonomic disturbance, sleep disturbance, and depression [26]. The severity of parkinsonism has been evaluated by using the symptom-based unified Parkinson's disease rating scale (UPDRS) [27] that covers various aspects of symptoms in patients with PD; it is an accurate tool to evaluate motor status of patients with PD based on its clinimetric properties. However, the scoring is not free from interrater variance or the fluctuation of the symptoms and may not adequately reflect disease severity [1]. The SL, SE, and 5 h seem to be useful for sleep dysfunction evaluations. Comella et al. [28] used these parameters to assess the effect of pergolide administered at nighttime on actigraphic measures of SE and sleep fragmentation. Other researchers [17] evaluated the therapeutic effect of one traditional Chinese medicine (TCM), Yang-Xue-Qing-Nao granules (YXQN), on sleep dysfunction in PD. The DA, EA, and NA with Parkinson's disease sleep scale (PDSS) and UPDRS scores quantitatively demonstrated YXQN sleep effect in PD without many side effects. The PLE might be one sensitive tool for the quantitative evaluation of physical activity in patients with PD. Lower PLE for local maxima or minima has been used to evaluate the effects of TCM in treating motor syndromes of PD [29]. They found activity during sleep time was markedly decreased and the local PLE value was significantly decreased during sleep time as well as during awake time. Improvement of wake-sleep rhythm, which is determined by browsing the day to day recorded activities, is likely a reflection of an improvement in PD. It could demonstrate that TCM may have beneficial effects on ADL by ameliorating the symptoms resulting from parkinsonism without exacerbating the L-dopa-induced adverse effects, as demonstrated in the improvement in scores of UPDRS Part IV. Researchers [12] have also evaluated the effect of 24-hour noisy galvanic vestibular stimulation (GVS) on PLE of daytime wrist activity and found that the PLE for the local maxima was significantly lower with the noisy GVS than with sham stimulation, which is suggestive of more frequent switching behavior from low to high levels of activity or less severe akinesia after GVS for patients with PD.

Pan et al. [7] used a longitudinal design in which physical activity and disease severity were assessed repeatedly during a 4-month interval, over a 3-year observational period, in patients with idiopathic PD. They found an increase in the maxima values of PLE and the UPDRS total score in PD patients and that there was a positive association between changes in maxima values and UPDRS scores. Changes in physical activity, as captured by actigraphy, are associated with increased severity in clinical symptoms of PD over time. This suggests that, when used in conjunction with the conventional UPDRS measure, an actigraphic measure of physical activity may provide clinicians with an adjunct measurement approach to monitor activity-based disease progression or responses to treatment in outpatient clinic settings.

## 4. Use in Evaluating the Severity of Sleep Disorders and Effects of Drugs

Actigraphy lacks the ability to distinguish between nocturnal activity due to wakefulness and activity due to other nocturnal sleep disturbances such as rapid eye movement (REM) sleep behavior disorder, periodic limb movements, or sleep apnea. A series of studies have demonstrated the utility of actigraphy and its application in assessing nocturnal activity. Nocturnal sleep disturbance may arise from these symptoms, and the demonstration of improvements may indicate that sleep disturbance can be treated according to alternative theories [30]. How should we assess REM sleep behavior disorder, periodic limb movements, or sleep apnea? The SL, SE, and 5 h of AMI records as well as clinical sleep scores would be potentially suitable tools to evaluate sleep disturbance. Qi et al. evaluated the effects of one TCM compound on patients with geriatric chronic obstructive pulmonary disease (COPD) [31]. The daily profiles and biorhythms, SL, SE, awake time activity, and 5 h of actigraphic records were markedly better in the TCM treatment group than in the control group. This indicates that evaluating actigraphic records may provide useful objective information for controlling COPD in outpatient clinics and for evaluating sleep disorders drugs.

Jiang and Wu [32] in order to explore the relationship between heart rate variability (HRV) and sleep phases, DFA and autoregressive (AR) model spectrum estimation were used to analyze R-R interval (RRI) sequence of healthy subjects. They detected long-range correlation in RRI sequence in different sleep phases and found that scaling exponents had more significant differences than spectral parameters in distinguishing different sleep phases. They demonstrated that DFA might be a better analysis method to quantify HRV in different sleep stages. Lee et al. [33] used DFA to analyze the signal of electroencephalogram (EEG) in sleep apnea and found that the mean score of DFA of EEG is discriminated according to nonrapid eye movement (non-REM) and rapid eye movement (REM) and waken stage and gradually increased from stage 1 to stages 2, 3, and 4. The scaling exponent of DFA might indicate sleep quality.

Jean-Louis et al. [9] used Actigraph Data Analysis Software (ADAS) which allows visual inspection of activity patterns derived from several actigraphic variables by using computer algorithms, including total sleep time (TST) and SE. ADAS was initially optimized in individuals with no sleep disturbances, yielding a correlation coefficient of 0.97 and a measurement error of 12 min, relative to polysomnographic sleep estimates. A previous study [10] investigated whether actigraphy measurements and sleep logs exhibited the same relationship with an established circadian phase marker, urinary 6-sulphatoxymelatonin (aMT6s), in a study that lasted at least 4 weeks. They found that the actigraph recorded a shorter SL, advanced onset time, increased number, and duration of night awakenings, delayed offset, increased night sleep duration, and increased number and duration of naps compared with the subjective sleep logs. The methods agreed when assessing changes in sleep in relation to aMT6s rhythm in the subjects.

Chin et al. [11] estimated sleep duration at night by analyzing wrist actigraphy tracings in conjunction with a sleep diary in Japanese subjects. The respiratory disturbance index was calculated from both the actigraph and type 3 portable monitor (PM) data. The PM records were visually inspected and scored by at least 2 medical doctors specializing in respiratory medicine. Apnea and hypopnea were scored blind to other information, except for sleep/wake time estimated by actigraphy. Retrospective measurements of fasting blood parameters were obtained from the company's periodical inspection data. Subjects with severe obstructive sleep apnea (OSA) had significantly shorter sleep duration. Sleep duration in metabolic syndrome (Mets) subjects was also significantly shorter than in those without OSA. These findings suggest that, in Japan, physicians should take into account this high prevalence of severe OSA in patients with Mets. Sleep duration distinguished by actigraphy should be taken into consideration as an important factor in studies investigating the prevalence of severe OSA and Mets.

## 5. Using Actigraphy to Evaluate the Severity of Stroke Combined with Upper Limb Motor Function Disorder

During treatment in the rehabilitation of stroke patients, we always ignore the recovery of sleep quality as well as its impact on the daily lives of patients. Bakken et al. [34] evaluated the activity and sleep of 90 first-time stroke patients using wrist actigraphy. The recording periods included the first two weeks in hospital and at 6 months of follow-up. The results showed that, in the acute phase, activities of daily living had a strong correlation with sleep during the day. Strong pain and a lower physical function may be important factors affecting the activities of daily living both in the acute phase and the follow-up. Sleep patterns in the acute phase may influence the patients' activities of daily life functioning up to 6 months after stroke. The authors suggested that wrist actigraphy might be used to observe sleep quality and pain in the rehabilitation period in stroke patients.

Sun et al. [8] used actigraphy to measure the severities of patients with stroke or PD by 2 different specified methods, PLE and DFA, with the clinical scores obtained by Fugl-Meyer assessment (FMA) and functional independence measure (FIM) in patients with stroke or with UPDRS in PD. Linear regression was found between FMA or FIM and DFA and between PLE values and UPDRS, suggesting actigraphy records and analysis of its PLE or DFA might provide useful objective and specific information for controlling stroke or PD patients and monitoring drug administrations, respectively.

## 6. Using Actigraphy to Evaluate Psychiatric Disorders

Seasonal affective disorder (SAD) is a recurring mood disorder with symptoms of fatigue and diminished energy. Patients with SAD might have disturbance in circadian and hemicircadian rhythms. Ohashi et al. [25] investigated whether circadian and hemicircadian rhythms are alterations in fluctuations within shorter time scales by calculating the scaling exponent of daytime and nighttime activities of actigraph recordings of children with SAD. They found that children with SAD had larger scaling exponents compared to controls during daytime for periods with higher and lower activities, which means that healthy controls have more abrupt activity bursts and dips while children with SAD have more sluggish transitions in their diurnal and nocturnal activity levels.

Nakamura et al. [35] described how resting and active periods are interwoven throughout daily life by actigraphic analysis. Active period durations with a physical activity count successively above a predefined threshold, when rescaled with individual means, follow a universal stretched exponential (gamma-type) cumulative distribution with characteristic time, both in healthy individuals and in patients with major depressive disorder. This means that resting period durations below the threshold for both groups obey a scale-free power law cumulative distribution over two decades, with significantly lower scaling exponents in depression. They demonstrate universal distribution laws governing human behavioral organization, with a parameter altered in depression.

By using DFA analysis, Pan et al. [13] evaluated the severity of behavioral and psychological symptoms of dementia (BPSD) for vascular dementia (VD) from actigraphy records and compared the results with clinical scores such as neuropsychiatric inventory (NPI) and the behavioral pathology in Alzheimer's disease (BEHAVE-AD) rating scale. At the end of 24 weeks, the subscores of activity disturbances, diurnal rhythm disturbances, and anxieties and phobias in the BEHAVE-AD score and those of agitation, irritability, and sleep disorder in the NPI score were significantly increased compared with the first week as were the changes for EA in the DFA value. A linear correlation was observed between the changes in activity disturbances plus anxieties and phobias and those of DA and between the development of diurnal rhythm and those of EA, the vehement and autism scores and those of NA, and the difference in sleep disorder scores and those of EA, respectively. They demonstrated that analysis of DA, NA, and EA may reflect the degree of fluctuation of VD-BPSD and might provide a useful assessment of VD-BPSD accompanied by clinical scores for VD. Mahlberg et al. have used actigraphic records to detect psychomotor agitations in patients with cognitive decline and neuropsychiatric complications [36, 37]. They found that actigraphic measurements showed a tendency towards reduced motor activity in the rivastigmine treatment by the means of actigraphic recordings, and actigraphy may well come to play an important role in monitoring treatment success in BPSD.

Hu et al. [38] investigated how human motor control systems orchestrate complex scale-invariant patterns of activity over a wide range of time scales. They studied spontaneous daytime activity patterns in 13 young adults, 13 elderly people with early-stage AD matched with 13 elderly controls, and 14 very old people with late-stage AD matched with 12 very old controls. In young adults, they found that activity exhibited robust scale-invariant correlations across all tested time scales (minutes to 8 h). The scale-invariant correlations at 1.5–8 h declined with age and were significantly reduced in the elderly and very old controls. An age-independent AD effect further reduced the scale-invariant correlations at 1.5–8 h, leading to the greatest reduction of the scale-invariant correlations in very old people with late-stage AD, although age may represent a potential confounder affecting results based on DFA analysis of human motion actigraphic records when applied to case-control studies. Thus, aging and AD significantly attenuate the scale invariance of activity fluctuations over multiple time scales.

Kim et al. [39] examined the variations in momentary symptoms of depressive mood, anxious mood, and fatigue and investigated covariant properties between the symptoms, and local statistics of locomotor activity were also detected by using an actigraphy. Multilevel modeling analysis confirmed convergent associations by showing positive correlations among momentary symptoms. The increased intermittency of locomotor activity, characterized by a combination of reduced activity with occasional bursts, appeared concurrently with the worsening of depressive mood, indicating that the temporal variations in the momentary symptoms are not random but reflect the underlying changes in psychophysiological variables in daily life. Their findings on the concurrent changes in depressive mood and locomotor activity may contribute to the continuous estimation of changes in depressive mood and early detection of depressive disorders.

## 7. Discussion and Conclusion

An actigraph is easy to wear, has less impact on a patient's daily life, and can reduce the subjective deviation of a rating scale. Although it has potential limits and minimal detectable change computation to obtain clinical relevant effects due to therapeutic interventions of such proposed tools in terms of accuracy, it may assist physicians in obtaining more objective and quantitative indexes to guide clinical treatments or studies, improving the reliability of clinical evaluations and furthering our understanding of the properties of neurological or psychopathic disorders by using physical activity as integrative medicine. These methods may be useful for the evaluation of disease progression and the efficacy of new drugs. We expect that researchers may develop more sensitive and specified methods to quantitatively and objectively assess more neurological or psychopathic disorders.
